# Supplementary material for: Nuclear spin coupling crossover in dense molecular hydrogen
Source: Nat Commun. 2020 Dec 10;11:6334. doi: 10.1038/s41467-020-19927-y (PMC7728769; doi:10.1038/s41467-020-19927-y)
Supplement: Supplementary file 1 — Supplementary Information [file 41467_2020_19927_MOESM1_ESM.pdf]

**Nuclear Spin Coupling Crossover in Dense Molecular Hydrogen**

**Supplementary Material**

Thomas Meier[1*], Dominique Laniel[2], Miriam Pena-Alvarez[3], Florian Trybel[1], Saiana Khandarkhaeva[1], Alena Krupp[1], Jeroen Jacobs[4], Natalia Dubrovinskaia[2], Leonid Dubrovinsky[1]

1) Bayerisches Geoinstitut, University of Bayreuth, Bayreuth, Germany
2) Material Physics and Technology at Extreme Conditions, Laboratory of Crystallography, University of Bayreuth, Bayreuth, Germany
3) Centre for Science at Extreme Conditions and School of Physics and Astronomy, University of Edinburgh, Edinburgh, United Kingdom
4) European Synchrotron Radiation Facility (ESRF), Grenoble Cedex, France

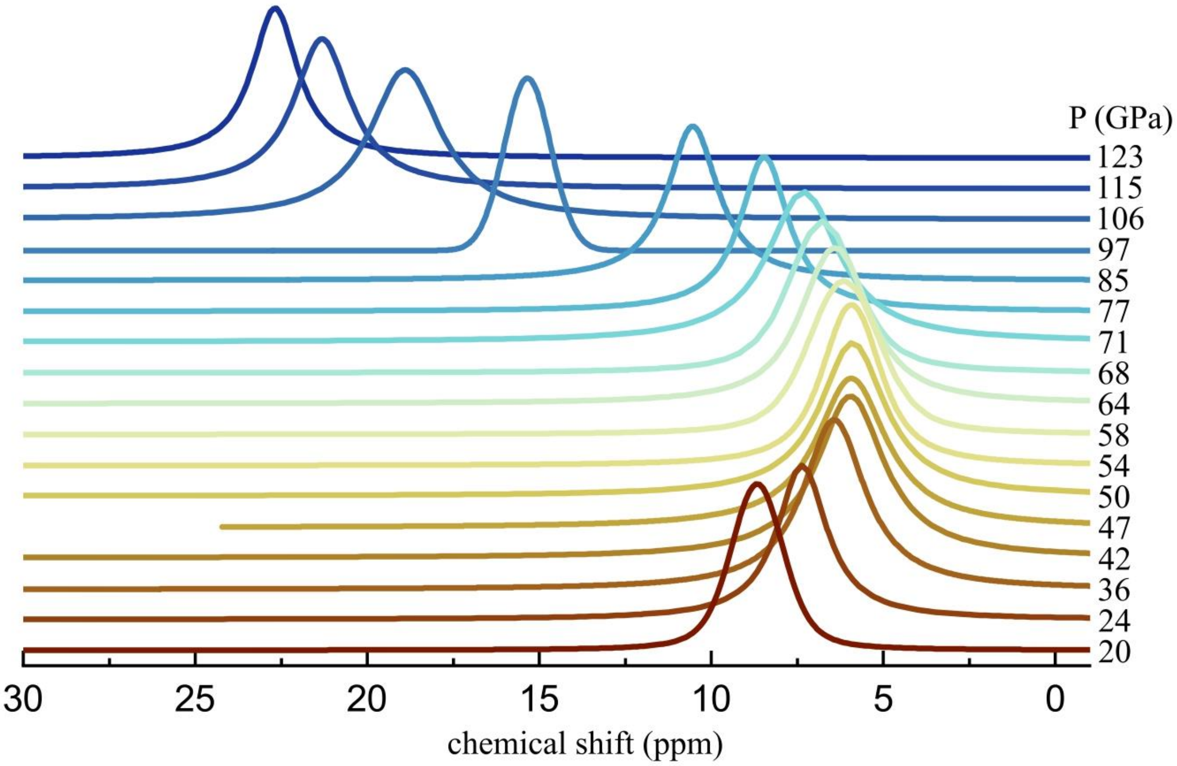


*Figure 1: **High Resolution $^1$H-NMR spectra of molecular hydrogen**. Spectra were recorded under compression using a Lee-Goldburg pulse scheme for homonuclear decoupling.*

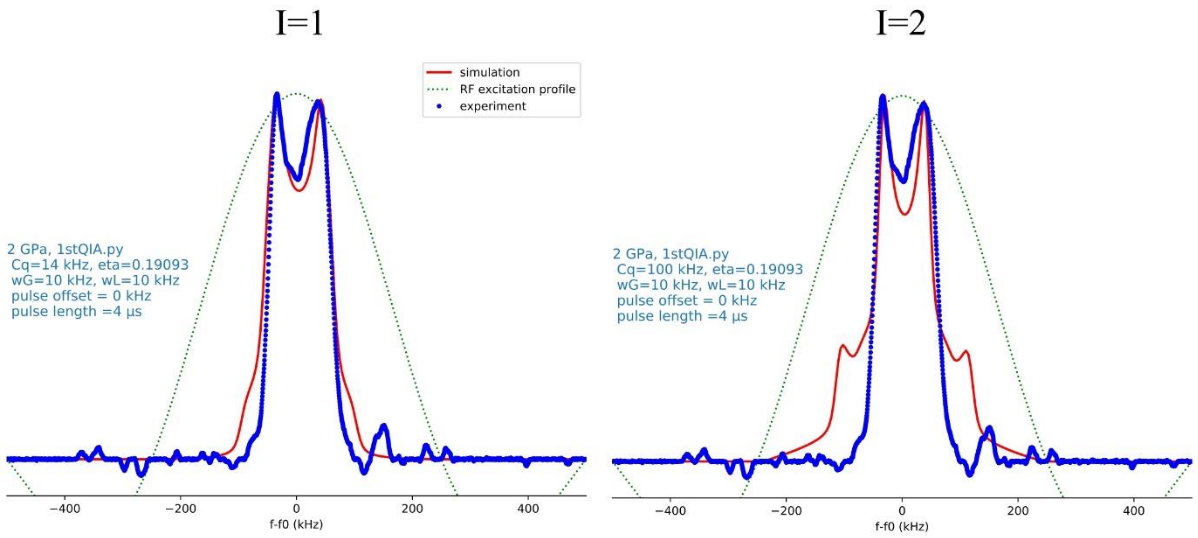


*Figure 2: $^{14}$N-NMR spectra of molecular nitrogen at pressures of about 3 to 4 GPa. Left) simulation of a I=1 lineshape. Right) simulation of a I=2 lineshape.*
